# Supplementary material for: Advances in Integrated Antimicrobial Resistance Surveillance and Control Strategies in Asia-Pacific Economic Cooperation Economies: Assessment of a Multiyear Building Capacity Project
Source: Antibiotics (Basel). 2022 Jul 29;11(8):1022. doi: 10.3390/antibiotics11081022 (PMC9405055; doi:10.3390/antibiotics11081022)
Supplement: Supplementary file 1 [file antibiotics-11-01022-s001.zip › antibiotics-1600994-supplementary.pdf]

File S1. Quick Survey for the Analysis of the Integrated Surveillance of Antimicrobial Resistance (AMR) in each Economy.

Introduction

Project Summary

Coordinated Research Initiative for the Implementation of Antimicrobial Resistance Control Strategies

In support of APEC's 2014 Rank 1 priority of promoting regional economic integration via free, open trade and investment through standards, conformity assessment, technical regulation and regulatory cooperation, Chile is seeking APEC funding for a Workshop that will allow the creation of a coordinated initiative between APEC economies in antimicrobial resistance control strategies.

The activity will focus on Antimicrobial resistance (AMR) present in microbial strains isolated from animals, environment and food, in order to lay the foundations and economies' needs for establishing an integrates Antimicrobial Resistance Surveillance System in each economy in the short term. Economies with more experience in the subject and ongoing surveillance systems can contribute with expertise and sharing training materials for the workshop. OIE and the WHO are key stakeholder of this initiative, considering the tripartite effort that FAO/OIE/WHO have made in order to develop a joint action plan on AMR. The Codex Alimentarius Commission, Intergovernmental Task Force of Antimicrobial Resistance science-based guidance's will also be considered in the project implementation. The project consists of a workshop that will aim to extend the capabilities and expertise of academia and national researchers, and awareness among producers and industry. The Workshop will be held in Santiago, Chile, during 28-30 of October, 2015. The outcome of this Workshop will include a capacity building training module on antimicrobial resistance for use by all APEC economies. Also, a final report of the advances and recommendations for economies will be delivered.

Project Objectives

- Enhance awareness, skills and knowledge in the field of AMR (focused on bacterial antimicrobial resistance), as a worldwide emerging issue in public health. This will be accomplished by sharing the experiences of the different APEC economies and AMR experts that attend the workshop.

- Strengthen technical competence of APEC economies´ representatives working in the areas related with AMR. This objective will be addressed by the lectures given by the invited experts that will participate in the workshop; these experts will provide APEC economies´

representatives with the tools to address this public health problem in order to work towards harmonized and standardized methodologies.

- Increase understanding of the control strategies of emergence and spread of AMR that have been used by other economies in the region and international organizations in order to improve economies' control measures and regulatory oversight of this hazard.
- Build competency and capacity in AMR surveillance through knowledge transfer between APEC economies with ongoing surveillance programs and other developing APEC economies that do not have an ongoing surveillance program on this subject.

We suggest that this survey should be completed in a multidisciplinary way, by members of different field of the health area.

Survey

**Member Economy**	
Member Economy Name	
**Details of the people who completed the survey**	
Name:	
Academic degrees/diploma:	
Position:	
Institution:	
Phone number:	
E-mail:	
**¿Did you work with other institutions to complete thus survey?**	
Institution which completed the survey	□ Ministry of Health □ Ministry of Agriculture □ Ministry of Foreign Affairs □OPS/Economy<br>□ OPS Region □WHO/Geneva □ Consultant<br>□Other_________________
Date of completion (DD/MM/YY)	/ /

General Information

In the national context regarding the issue of antimicrobial resistance and appropriate interventions to lessen their impact, there are needs and actions that have to take place, but they must be based on knowledge.

1) List the 5 bacterial zoonotic diseases and/or foodborne diseases that represent the major challenge for public health in your economy.

1.
2.
3.
4.

2) List the 5 bacterial infectious diseases that represent the major challenge for public health in your economy. (You are allowed to repeat from the above)

1.
2.
3.
4.
5.

3) How would you describe the awareness of antimicrobial resistance in human infectious diseases in your economy?

☐ High ☐ Medium ☐ Low ☐ None ☐ Not known

4) How would you describe the awareness of agriculture/veterinary use of antimicrobials and their contribution to AMR in human infectious diseases in your economy?

☐ High ☐ Medium ☐ Low ☐ None ☐ Not known

5) What would be the level of awareness in each of these different groups?

Politicians	☐ High	☐ Medium	☐ Low	☐ None	☐ Not known
Health Staff	☐ High	☐ Medium	☐ Low	☐ None	☐ Not known
Veterinary Staff	☐ High	☐ Medium	☐ Low	☐ None	☐ Not known
Farmworkers	☐ High	☐ Medium	☐ Low	☐ None	☐ Not known
Pharmaceutical Industry	☐ High	☐ Medium	☐ Low	☐ None	☐ Not known
Agroindustry	☐ High	☐ Medium	☐ Low	☐ None	☐ Not known

General Public	☐ High	☐ Medium	☐ Low	☐ None	☐ Not known
Mass Media	☐ High	☐ Medium	☐ Low	☐ None	☐ Not known
Academics	☐ High	☐ Medium	☐ Low	☐ None	☐ Not known
Governmental Institutions	☐ High	☐ Medium	☐ Low	☐ None	☐ Not known

6) Of the following matters: Which ones are of most concern in addressing the issue of antimicrobial resistance in your economy?

1. ☐ Lack of Antibiotic (AB) registry
2. ☐ Lack of alternatives to AB
3. ☐ Use of AB as growth promotors
4. ☐ Lack of legislation to monitor or control use of AB
5. ☐ Lack of audit of AB use at farm or veterinary clinics
6. ☐ Nonexistence of AMR surveillance programs (in human health)
7. ☐ Nonexistence of AMR surveillance programs (in food/animals)
8. ☐ Use of critically important AB for humans used in Agroindustry
9. ☐ Lack of prescription for veterinary AB sales
10. ☐ Lack of technical knowledge of AMR
11. ☐ Lack of resources to take actions on this issue

7) Does a National Regulatory Authority (NRA) in charge of the registry and authorization of drugs for human use exist in your economy? ☐ Yes ☐ No ☐ Not known

Name it:

8) Does a List of Essential Drugs (LED) exist in your economy? ☐ Yes ☐ No ☐ Not known
If it does, please include an annex or internet link to the most recent report.

9) Does a government agency in charge of the registry and authorization of drugs for veterinary use exist in your economy? ☐ Yes ☐ No ☐ Not known

Name it:

10) Does this control take place with traceability (series control throughout the food chain) program for the use of these drugs? ☐ Yes ☐ No ☐ Not known

11) Does a program for the control of veterinary drug residues in food and/or the environment exist in your economy? ☐ Yes ☐ No ☐ Not known

Name the institution in charge:

12) The list of authorized veterinary drugs in your economy corresponds to:

a. ☐ Own legislation
b. ☐ Own legislation in line with Codex
c. ☐ Only Codex
d. ☐ Don't have

13) In your economy, do national standards or guidance to address AMR exist (i.e. hospital infection control, prudent use guidelines etc? ☐ Yes ☐ No ☐ Not known

If yes, please include an annex or internet link to the standards.

14) In your economy, by law: ¿Do AMR surveillance programs exist? ☐ Yes ☐ No ☐ Not known

If yes, please include an annex or internet link to the surveillance programs.

15) In your economy, do national standards to approach AMR exist? ☐ Yes ☐ No ☐ Not known

16) If your previous answer is yes, please indicate (mark with an X if isolation from these samples is performed):

Bacterial Genus	Isolated from humans	Isolated from animals	Isolated from food

17) In your economy: ¿Which institution is responsible for AMR surveillance?

Indicate if this institution is of:
☐ Governement
☐ University
☐ Private
☐ Other

18) Does a report of epidemiological surveillance (5 years old or less) in humans exist in your economy?
☐ Yes ☐ No ☐ Not known
If it does, please include an annex or internet link to the most recent report.

19) ¿Are concrete actions taken with the results of surveillance reports on AMR in humans?

☐ Yes ☐ No ☐ Not known
If it does, please include an annex or internet link.

20) Does a report of epidemiological surveillance (5 years old or less) on AMR in animals exist in your economy?
☐ Yes ☐ No ☐ Not known
If it does, please include an annex or internet link to the most recent report.

21) ¿ Are concrete actions taken with the results of surveillance reports in animals?
☐ Yes ☐ No ☐ Not known
If it does, please include an annex or internet link.

It is necessary to address the problem of antimicrobial resistance from a multisectoral perspective, integrating the vision the main areas as human health, animal and food production, in order to address the problem effectively.

22) Is there a mechanism, on a national level, which coordinates AMR activities in your economy?
☐ Yes ☐ No ☐ Not known

23) If you previous answer is yes, indicate the institution and the position of the person in charge.

24) If you answered yes in question n° 22: Who is represented in the coordination mechanism? Mark the institutions and specialty in charge.

Institutions	***Technical specialities***
☐ Not known	☐ Not known
☐ Ministry of Health	☐ Infectious Disease
☐ Ministry of Agriculture	☐ Tuberculosis
☐ Regulatory Authority for Human drugs	☐ Pharmacy
☐ Regulatory Authority for Animal drugs	☐ Microbiology
☐ Food Safety Authority	☐ Infection Prevention
☐ Epidemiological Surveillance Coordinator	☐ Epidemiology
☐ Hospitals (on a National level)	☐ Veterinary
☐ Hospitals (on a sub-national level)	☐ Animal Husbandry
☐ Patient groups and/or civil society groups	☐ Aquaculture
☐ Professional Associations	☐ Food Safety
☐ Networks	☐ Other (please specify)
☐ Universities	______________________
☐ Research Institutions	
☐ Other (please specify)	
____	

25) If your previous answers were negative indicate a possible institution that could take care of coordinating a AMR national surveillance system:

26) Does the national surveillance system for AMR receive any funding?
☐ Yes ☐ No ☐ Not known
Is this founding: ☐ Public ☐Private ☐Public/Private

27) Does a management plan for the national surveillance system for AMR exist in your economy?
☐ Yes ☐ No ☐ Not known

28) Is the AMR management plan active? (for ex: were there any meetings in the last year?
☐ Yes ☐ No ☐ Not known

29) Does the management plan include indicators and/or goals? ☐ Yes ☐ No ☐ Not known

30) Is there a national report on AMR progress (updated in the last 5 years)?
☐ Yes ☐ No ☐ Not known

31) Is there an official National Reference Laboratory (NRL) (or some other key public laboratory performing some or all of the typical tasks of NRL) for AB susceptibility testing?
☐ Yes ☐ No ☐ Not known
If your answer is yes, please indicate the name of the laboratory:

32) Does the NRL (or other key laboratory) participate in a program of External Quality Assessment for antimicrobial susceptibility testing? ☐ Yes ☐ No ☐ Not known

Optimal use of antimicrobials is important to minimize the emergence and spread of antimicrobial resistance and also prolongs the service life of antimicrobials.

33) Does your legislation allow the sale of antimicrobials for human use without a prescription?
☐ Yes ☐ No ☐ Not known

34) Does your legislation allow the sale of antimicrobials for animal use without a prescription?
☐ Yes ☐ No ☐ Not known

35) If your answer is negative, what mechanisms does the National Regulatory Authority use to implement the use of AB only with prescription?

☐ Sale with prescription (without stamp or retention)
☐ Stamp on the prescription (so you can’t use it again)
☐ Prescription retention
☐ Central computerized system
☐ No actions are taken

36) Have there been any public education campaigns on the correct use of antimicrobials for human use in the last two years? ☐ Yes ☐ No ☐ Not known

37) Have there been any public education campaigns on the correct use of antimicrobials for animal use in the last two years? ☐ Yes ☐ No ☐ Not known

38) Is there any legislation to control the use of AB in animal husbandry?
☐ Yes ☐ No ☐ Not known
If so, please include internet link or attach the document

39) Are there standards for the monitoring of AMR in animal husbandry environments?
☐ Yes ☐ No ☐ Not known

40) Does the National Regulatory Authority uses any mechanism to implement the requirements for the rational use of antimicrobials in the field of animal husbandry? ☐ Yes ☐ No ☐ Not known

The need for antimicrobials can also be reduced with good control of infections in humans and in the veterinary field.

41) Is there a national program of mandatory reporting of infectious diseases in human patients?
☐ Yes ☐ No ☐ Not known

42) If you previous answer is yes, are there specific measures to control the AMR included in the mandatory reporting of infectious diseases in human patients? ☐ Yes ☐ No ☐ Not known
List the bacterias included in this program:

43) What proportion of tertiary hospitals have control programs on AMR of mandatory reporting of infectious diseases in human patients?

☐ 100%
☐ >50%
☐ 50%
☐ <50%
☐ 0%
☐ Not known

Innovation in all areas for the development of new approaches, tools and medicines are required to contain the emergence and spread of AMR.

44) Is there in the economy "research and development" (R & D) related to AMR? If there is, please identify the area of R & D (i.e. epidemiological studies, assessment of resistance mechanisms). ☐ Yes ☐ No ☐ Not known

45) If your answer to the previous question is yes, which institutions carry out the research?

☐ Governement ☐ University ☐Private ☐Public/Private

46) Prioritize 5 actions that you consider necessary to generate an integrated AMR surveillance (humans, animals and food) plan on AMR in your economy.

Supplementary Table S1: AMR surveillance or national action plans of the participating countries.

Country	AMR surveillance or national action program	Release date	Source
Australia	Australia's National Antimicrobial Resistance Strategy	2015	https://www.amr.gov.au/resources/australias-national-antimicrobial-resistance-strategy-2020-and-beyond
Canada	Canadian Integrated Program for Antimicrobial Resistance Surveillance (CIPARS)	2002	https://www.canada.ca/en/public-health/services/surveillance.html
United States	The National Antimicrobial Resistance Monitoring System for Enteric Bacteria (NARMS)	1996	https://www.cdc.gov/narms/index.html#:~:text=The%20NARMS%20program%20at%20CDC,infections%20differ%20from%20susceptible%20infections.
Hong Kong	Hong Kong Strategy and Action Plan on Antimicrobial Resistance	2017	https://www.chp.gov.hk/files/pdf/amr_action_plan_eng.pdf
Japan	National Action Plan on Antimicrobial Resistance (AMR)	2016	https://www.mhlw.go.jp/file/06-Seisakujouhou-10900000-Kenkoukyoku/0000138942.pdf
New Zealand	New Zealand Antimicrobial Resistance Action Plan	2017	https://www.health.govt.nz/our-work/diseases-and-conditions/antimicrobial-resistance/new-zealand-antimicrobial-resistance-action-plan
Chile	Chilean National Plan on Antimicrobial Resistance	2017	https://diprece.minsal.cl/wp-content/uploads/2022/03/PLAN-RAM-ENGLISH-VERSION.pdf
Indonesia	National Action Plan on Antimicrobial Resistance Indonesia	2016	https://www.flemingfund.org/wp-content/uploads/59f148b0482cda160087e29b9a5a21a0.pdf
Malaysia	Malaysian Action Plan on Antimicrobial Resistance (MyAP-AMR)	2017	https://www.moh.gov.my/moh/resources/Penerbitan/Garis%20Panduan/Garis%20panduan%20Umum%20(Awam)/National_Action_Plan_-_FINAL_29_june.pdf
Mexico	National Strategy for Action against Antimicrobial Resistance	2018	https://dof.gob.mx/nota_detalle.php?codigo=5525043&fecha=05/06/2018#gsc.tab=0
Peru	National action plan to combat antimicrobial resistance	2017	https://www.who.int/publications/m/item/peru-national-action-plan-to-combat-antimicrobial-resistance
Philippines	The Philippine Action Plan to Combat Antimicrobial Resistance	2015	https://cdn.who.int/media/docs/default-source/antimicrobial-resistance/amr-spc-npm/nap-library/philippine-national-action-plan-on-amr-2019-2023-final.pdf?sfvrsn=8bbe1fdb_1
Thailand	Thailand's National Strategic Plan on Antimicrobial Resistance	2017	https://amrthailand.net/English
Vietnam	Not available		
